# Prognostic Value of Admission Glucose and Extended Lipid Profile in Patients with Crimean‐Congo Hemorrhagic Fever

**DOI:** 10.1002/jmv.70918

**Published:** 2026-04-08

**Authors:** Yasemin Çakır Kıymaz, Cihad Baysal, Caner Öksüz, Seyit Ali Büyüktuna, Nazif Elaldı

**Affiliations:** ^1^ Department of Infectious Diseases and Clinical Microbiology, Faculty of Medicine Sivas Cumhuriyet University Sivas Turkey

**Keywords:** blood glucose, Crimean‐Congo hemorrhagic fever, dyslipidemia, prognosis

## Abstract

Crimean‐Congo hemorrhagic fever (CCHF) is a life‐threatening viral hemorrhagic fever with a highly variable clinical course. While viral and inflammatory markers determining disease severity are well defined, the impact of the host's metabolic status on CCHF severity remains largely unexplored. The aim of this study is to investigate the relationship between metabolic syndrome components, lipid profile, and disease severity in patients with CCHF. This single‐center, prospective, case‐control study included 74 polymerase chain reaction (PCR)‐confirmed CCHF patients and 31 healthy controls. Patients were stratified into mild and moderate‐severe disease groups based on clinical findings and severity grading scores. Metabolic parameters, including body mass index (BMI), fasting blood glucose (FBG), and lipid profile, were compared between patient and control groups as well as according to disease severity. The patient and control groups were similar in terms of age, gender, BMI, and FBG levels. Low‐density lipoprotein cholesterol (LDL‐C), levels were significantly lower in CCHF patients compared to the control group (*p* = 0.042), while high‐density lipoprotein cholesterol (HDL‐C), levels were lower with borderline significance (*p* = 0.051). Patients in the moderate‐severe disease group had significantly higher BMI (*p* = 0.010), FBG (*p* < 0.01), and triglyceride levels (*p* = 0.033) compared to those with mild disease. ROC analysis demonstrated that an FBG cut‐off value of > 105 mg/dL had the highest discriminatory power in predicting severe disease (AUC: 0.761). In multivariate logistic regression analysis, FBG (OR: 1.026; 95% CI: 1.007–1.045; *p* = 0.007) was identified as independent factors associated with disease severity.

Metabolic parameters, particularly FBG and triglyceride levels, are significantly associated with disease severity in CCHF. Evaluation of the metabolic status at admission may contribute to early risk stratification and the improvement of clinical management in CCHF patients.

## 1 Introduction

Crimean‐Congo Hemorrhagic Fever (CCHF) is a tick‐borne zoonosis characterized by a wide geographical distribution, high mortality rates, and significant threats to public health [1]. The clinical course of the disease exhibits a broad spectrum, ranging from asymptomatic infections to fatal presentations characterized by widespread hemorrhages, shock, and multiorgan failure [2]. In this process, the fundamental pathophysiological mechanism determining disease severity is widespread endothelial damage resulting from the direct effect of the virus and an uncontrolled immune response [3]. However, this distinct diversity in the clinical picture, despite similar viral loads, cannot be explained by viral factors alone; it points to the role of the host's vascular and metabolic status in the prognosis of the disease [4].

In this context, host‐related metabolic factors specifically glucose metabolism and lipid profiles possess critical mechanisms that intersect with CCHF pathogenesis. Metabolic disturbances, such as hyperglycemia and dyslipidemia, are known to contribute to chronic systemic inflammation and baseline endothelial dysfunction [5]. Hyperglycemia and altered lipid levels can predispose the organism to vascular damage by disrupting vascular structure through oxidative stress and impaired nitric oxide bioavailability [6].

The convergence of acute endothelial destruction caused by CCHF and the pre‐existing vascular stress associated with impaired metabolic parameters may theoretically create a synergistic effect that aggravates disease severity. Despite this, most studies in the literature regarding CCHF prognosis have focused on acute viral markers and inflammatory cytokines [7, 8], while the number of studies investigating the role of host metabolic status such as admission lipid profile and blood glucose on disease severity remains limited^9.^

This study aims to investigate the prognostic value of admission metabolic markers, specifically fasting blood glucose (FBG) and an extended lipid profile (including LDL‐C, HDL‐C, triglycerides, and total cholesterol), in patients diagnosed with CCHF compared to a healthy control group. By elucidating the relationship between these metabolic markers and disease severity, our study seeks to offer new perspectives for risk assessment and address an existing gap in the literature regarding the impact of host metabolic background on CCHF prognosis.

## 2 Materials and Methods

### 2.1 Study Population

The study was designed as a prospective, observational case‐control study. The study included 74 patients with Polymerase Chain Reaction (PCR)‐confirmed CCHF who were followed up at the Sivas Cumhuriyet University Faculty of Medicine Hospital Infectious Diseases Clinic between June 30 and September 15, 2025. The control group consisted of 31 healthy individuals with similar age, gender and BMI distributions who did not have active infections, chronic inflammatory diseases, or known malignancies. Demographic characteristics such as age, gender, place of residence, occupation, history of tick bite, involvement in animal husbandry, smoking, and alcohol use were recorded. Clinical data evaluated included symptoms at presentation, time from symptom onset to hospital admission, length of hospital stay, intensive care unit (ICU) requirement, and mortality data. Patients were divided into two groups based on disease severity: mild and moderate‐severe. Metabolic parameters and laboratory values were compared between these groups. The Severity Grading Score (SGS) at admission was used for severity assessment [9].

### 2.2 Inclusion and Exclusion Criteria

Patients aged ≥ 18 years with PCR‐confirmed CCHF and complete clinical and laboratory data at admission were included in the study. To ensure that the observed metabolic and inflammatory changes were directly attributable to the acute phase of CCHF, several exclusion criteria were applied to minimize confounding factors. Specifically, individuals with a documented medical history of diabetes, thyroid dysfunction, or dyslipidemia, as well as those with chronic liver or kidney failure, malignancy, or autoimmune disease, were excluded. Pregnant and lactating women were also not included. Furthermore, to avoid secondary influences on lipid metabolism and immune response, patients receiving oral antidiabetics, insulin, lipid‐lowering agents (e.g., statins), systemic corticosteroids, or immunosuppressive drugs were excluded, as were individuals with a history of chronic alcoholism in both the patient and control groups. This rigorous selection process was specifically designed to isolate the acute impact of CCHF infection itself on lipid metabolism and metabolic parameters. By excluding patients with pre‐existing lipid disorders or those on pharmacological treatments, we aimed to ensure that the findings reflected the clinical course and severity of the disease rather than chronic baseline conditions or medication effects.

### 2.3 Metabolic and Laboratory Parameters

Venous blood samples were collected from all patients within the first 24 h of hospital admission (Day 1) after an 8‐h fast. Serum total cholesterol, high‐density lipoprotein cholesterol (HDL‐C), and triglyceride levels were measured using automated enzymatic colorimetric methods. Low‐density lipoprotein cholesterol (LDL‐C) was directly measured using a homogeneous enzymatic assay, instead of being calculated via the Friedewald formula.

Recorded metabolic parameters included BMI, fasting blood glucose (FBG), BMI was calculated by dividing weight in kilograms by the square of height in meters (kg/m^2^). Laboratory parameters included complete blood count (CBC), aspartate aminotransferase (AST), alanine aminotransferase (ALT), blood urea nitrogen (BUN), creatinine, C‐reactive protein (CRP), lactate dehydrogenase (LDH), creatine phosphokinase (CPK), ferritin, d‐dimer, fibrinogen, prothrombin time (PT), activated partial thromboplastin time (aPTT), INR, and interleukin‐6 (IL‐6) levels.

### 2.4 Statistical Analysis

Statistical analyses were performed using SPSS (IBM SPSS Statistics for Windows, Version 23.0; IBM Corp., Armonk, NY, USA), and data visualization was conducted using GraphPad Prism version 8.3.0 (San Diego, CA, USA). The distribution of continuous variables was evaluated using the Kolmogorov–Smirnov test. Continuous variables with normal distribution were expressed as mean ± standard deviation, while those without normal distribution were expressed as median (1–3 quartiles). Categorical variables were presented as counts and percentages. For comparisons between two groups, the independent samples t‐test was used for normally distributed data, the Mann–Whitney U test for non‐normally distributed data, and the Chi‐square or Fisher's exact test for categorical variables.

The performance of metabolic parameters in predicting disease severity was evaluated using Receiver Operating Characteristic (ROC) analysis, and optimal cut‐off values were determined. Logistic regression analysis was performed to identify independent factors associated with severe disease. Correlation analysis was used to evaluate the relationship between metabolic parameters and indicators reflecting inflammation and disease severity. Due to the non‐normal distribution of variables, the Spearman rank correlation test was used. Correlation coefficients (r) and *p*‐values were reported. The statistical significance level was set at *p* < 0.05.

### 2.5 Ethical Approval

This study was approved by the Sivas Cumhuriyet University Health Sciences Ethics Committee (Decision no: 2025‐06/45, Date: 12.06.2025). The study was conducted in accordance with the principles of the Declaration of Helsinki. Written informed consent was obtained from the patients.

## 3 Results

### 3.1 Study Population and Baseline Characteristics

The study included 74 patients diagnosed with CCHF and 31 healthy controls. The groups were well‐matched, with no significant differences observed regarding age, gender and BMI distribution (*p* = 0.694, *p* = 0.100 and *p* = 0.135, respectively; Table 1).

**Table 1:** Demographic, clinical and laboratory characteristics of patients and controls.

Variables	Patients (*n* = 74)	Controls (*n* = 31)	*p*‐value
**Demographic characteristics**			
Age (years)	47 (32–61.2)	49 (30–69)	0.694
Gender			
Male	57 (77)	19 (61.3)	0.100
Female	17 (23)	12 (38.7)	
BMI	26 ± 4.0	24.9 ± 4.2	0.135
Smoking status			
Yes	24 (32.4)	11 (35.5)	0.762
No	50 (67.6)	20 (64.5)	
Alcohol consumption			
Yes	4 (5.4)	0	0.317
No	70 (94.6)	31 (100)	
**Epidemiological data**			
Tick bite			
Yes	65 (87.8)		
No	9 (12.2)		
Residence			
Rural	68 (91.9)		
Urban	6 (8.2)		
Occupation			
Agriculture	53 (71.6)		
Housewife	7 (9.5)		
Others	14 (18.9)		
Animal husbandry			
Yes	61 (82.4)		
No	13 (17.6)		
**Clinical findings and course**			
Symptoms			
Fatigue	65 (87.8)		
Fever	64 (86.5)		
Myalgia	62 (83.8)		
Headache	42 (56.8)		
Nausea/vomiting	34 (45.9)		
Diarrhea	17 (23)		
Petechia/purpura/ecchymosis	9 (12.2)		
Bleeding on admission	2 (2.7)		
Symptoms onset to admission* (days)	2 (1–4)		
Type of bleeding			
Mucosal bleeding	8 (10.8)		
Gastrointestinal bleeding	3 (4.1)		
Genitourinary bleeding	3 (4.1)		
Alveolar hemorrhage	3 (4.1)		
Severity of diseases			
Mild	49 (66.2)		
Moderate‐severe	25 (33.8)		
Duration of hospitalization* (days)	7 (5–10)		
ICU admission	6 (8.1)		
Mortality	4 (5.4)		
**Laboratory parameters**			
Fasting blood glucose (mg/dL)	96 (89–115)	95 (84–100)	0.080
LDL cholesterol (mg/dL)	67 (50–86.5)	83 (62–98)	**0.042**
HDL cholesterol (mg/dL)	32.5 (28–43)	40 (31–48)	**0.051**
Total cholesterol (mg/dL)	122.5 (105.5–150.2)	136 (118–158)	0.066
Triglycerides (mg/dL)	102 (61.7–172)	97 (68–163)	0.902

*Note:* Numerical parameters following a normal distribution were compared using an independent *t*‐test. Non‐normally distributed numerical data were compared using the Mann‐Whitney U test. Categorical variables are presented as *n* (%), normally distributed numerical data as mean ± SD, and non‐normally distributed numerical data as median (1st‐3rd quartiles). *p* < 0.05 values are shown in **bold**. *p*‐values < 0.05 were considered statistically significant.

Abbreviations: BMI, body mass index; HDl, high‐density lipoprotein; ICU, intensive care unit; LDL, low‐density lipoprotein.

Among CCHF patients, the majority were male, resided in rural areas, and were engaged in agricultural activities. A history of tick bite was reported by most patients. The most frequent presenting symptoms included fatigue, fever, and myalgia, while bleeding signs at admission were rare. Based on disease severity, 66.2% of cases were classified as mild and 33.8% as moderate‐severe. Overall intensive care admission and mortality rates are summarized in Table 1.

### 3.2 Comparison of Metabolic Parameters in Patient and Control Groups

Analysis of the lipid profile revealed that LDL‐C levels were significantly lower in the CCHF patient group compared to the control group (*p* = 0.042). HDL‐C levels also appeared lower in patients, though this difference reached only borderline statistical significance (*p* = 0.051). No significant differences were detected between the patient and control groups concerning total cholesterol, triglycerides, BMI, or FBG levels (Table 1).

### 3.3 Metabolic and Laboratory Parameters According to Disease Severity

When compared based on disease severity, BMI values were significantly higher in the moderate‐severe CCHF group than in the mild group (*p* = 0.019). FBG and triglyceride levels were also markedly elevated in moderate‐severe patients (*p* < 0.001 and *p* = 0.033, respectively). In contrast, LDL‐C, HDL‐C, and total cholesterol levels did not differ significantly between these subgroups (*p* > 0.05; Table 2).

**Table 2:** Metabolic and laboratory parameters of patients with CCHF.

Variables	Reference range	Mild (*n* = 49)	Moderate‐severe (*n* = 31)	*p*‐value
**Metabolic parameters**				
BMI	18.5–24.9 (kg/m^2^)	25.2 ± 3.8	27.4 ± 4.0	**0.019**
Fasting blood glucose	< 100 (mg/dL)	91 (87.5–100)	124 (95.5–151.5)	**< 0.001**
LDL cholesterol	100 (mg/dL)	67 (49–85.5)	62 (54.5–93)	0.489
HDL cholesterol	> 40 (male)	33 (27.5–43)	32 (28–41)	0.571
	> 50 (female) (mg/dL)			
Total cholesterol	< 200 (mg/dL)	122 (105–141.5)	125 (106–154.5)	0.340
Triglycerides	150 (mg/dL)	75 (59.5–145.5)	152 (87–222)	**0.033**
**Clinical and viral parameters**				
Symptoms onset to admission (days)		2 (1–4)	3 (2–4.5)	**0.042**
Duration of hospitalization (days)		6 (5–8)	9 (5.5–11)	**0.025**
SGS on admission		1 (0–2)	2 (0.5–6)	**0.008**
Viral load (log₁₀ copies/mL)		6.06 (5.1–6.8)	7.04 (6.0–7.6)	**0.007**
**Hematological parameters**				
WBC	4–10 (10^9/L)	3.5 (2.5–4.8)	2.8 (1.8–5.5)	0.507
HGB	gr/dL	14 (13.2–15)	13.3 (12.3–15.1)	0.251
PLT	150–400 (10^9/L)	121 ± 44.8	80.1 ± 48.5	**0.001**
**Biochemical parameters**				
AST	< 40 U/L	34 (25.5–95)	95 (53–311)	**0.001**
ALT	< 40 U/L	27 (16.5–47.5)	41 (26–112.5)	**0.011**
BUN	6–120 (mg/dL)	15 (12.5–17.5)	17.3 (16–20.6)	**0.003**
Creatinine	0.7–1.2 (male)	0.9 (0.8–1.0)	1 (0.8–1.1)	0.254
	0.5–1.1 (female) (mg/dL)			
LDH	140–280 (U/L)	280 (220–379)	481 (308.5–713)	**< 0.001**
CPK	< 200 U/L	135 (100–313)	217 (135–503)	**0.045**
**Coagulation parameters**				
Fibrinogen	200–400 (mg/dL)	250 (223–307)	250 (190.5–278)	0.211
d‐dimer	< 5 (mg/L)	1.6 (0.86–3.45)	10.2 (2–31)	**< 0.001**
PT	11–13.5 (sec)	12.3 (11–12.7)	12.8 (11.4–13.9)	0.065
aPTT	25–35 (sec)	25.3 (23.4–26.9)	29.4 (26.3–32.8)	**< 0.001**
INR	0.8–1.2	1 (0.9–1.08)	1 (0.9–1.1)	**0.049**
**Inflammatory parameters**				
CRP	< 5 mg/L	9 (3.3–25)	34 (12.7–65.5)	**0.005**
Ferritin	30–400 (male)	408 (131–2485)	5819 (1817–15225)	**< 0.01**
	13–150 (female) (ug/L)			
IL‐6	< 7 (ng/L)	16.9 (0.3–52)	55 (24.1–136.5)	**0.001**

*Note:* Numerical parameters following a normal distribution were compared using an independent *t*‐test. Non‐normally distributed numerical data were compared using the Mann‐Whitney U test. Categorical variables are presented as *n* (%), normally distributed numerical data as mean ± SD, and non‐normally distributed numerical data as median (1st–3rd quartiles). *p* < 0.05 values are shown in **bold** *p*‐values < 0.05 were considered statistically significant.

Abbreviations: ALT, Alanine aminotransferase; aPTT, Activated partial thromboplastin time; AST, Aspartate aminotransferase; BMI: Body mass index; BUN, Blood urea nitrogen; CPK, Creatin kinase; CRP; C reactive protein; HDL, High‐density lipoprotein; HGB, Hemoglobin; IL‐6, Interleukin 6; INR, International normalized ratio; LDH, Lactate dehydrogenases; LDL, Low‐density lipoprotein; PLT, Platelets; PT, Prothrombin time; RBC, Red blood cells; SD, Standard deviation; WBC, White blood cells.

Patients in the moderate‐severe group experienced a longer duration from symptom onset to admission and an extended length of hospital stay (*p* = 0.042 and *p* = 0.025, respectively). Regarding laboratory findings, the moderate‐severe group exhibited significantly lower platelet counts, while markers such as AST, ALT, BUN, CRP, LDH, CPK, d‐dimer, aPTT, ferritin, and IL‐6 were significantly elevated (Table 2).

### 3.4 ROC Analysis for Predicting Severe Disease

ROC analysis was performed to evaluate the performance of metabolic parameters in predicting disease severity. Among the variables, FBG exhibited the highest discriminatory power (AUC: 0.761), followed by BMI (AUC: 0.696) and triglyceride levels (AUC: 0.652). Detailed cut‐off values, sensitivities, and specificities for these parameters are provided in Table 3.

**Table 3:** ROC analysis results for variables in predicting severe disease.

	Cut‐off	AUC	Sen%	Spe (%)	LR +	LR‐	PPV	NPV
Fasting blood glucose	> 105	0.761	64 (43–82)	84 (70–93)	3.9 (2–8)	0.4 (0.3–0.7)	67 (50–80)	82 (73–89)
BMI	> 26.5	0.696	64 (43–82)	65 (50–78)	1.8 (1.1–3.0)	0.5 (0.3–1.0)	49 (37–60)	78 (67–86)
Triglycerides	> 170	0.652	48 (28–69)	86 (73–94)	3.4 (1.5–7.5)	0.6 (0.4–0.9)	63 (44–79)	76 (69–83)

Abbreviation: BMI, body mass index.

### 3.5 Regression Analysis

**Figure 1:**
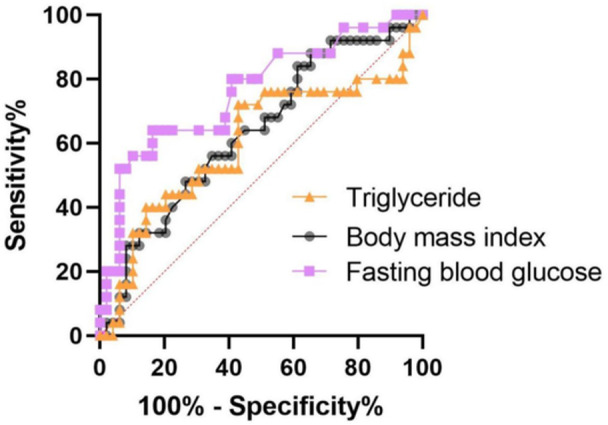
Comparison of ROC curves for FBG, BMI, and triglycerides in predicting mild‐moderate versus severe CCHF.

To identify independent predictors of severe disease, a logistic regression analysis was conducted. FBG was identified as a significant independent predictor of disease severity (OR: 1.026; 95% CI: 1.007–1.045; *p* = 0.007), even after adjusting for potential confounders (Table 4, Figure 1).

**Table 4:** Results of regression analyses for severe disease.

Parameters	Wald's x Step 1 (Initial)	*p*‐value	Wald's x	*p*‐value	OR (%95 Cl)
		Step 1 (Initial)	Step 4 (Final)	Step 4 (Final)	
BMI (kg/m^2^)	0.604	0.437			
Fasting blood glucose (mg/dL)	4.028	0.045	7.315	0.007	1.026 (1.007–1.045)
Triglycerides (mg/dL)	2.237	0.135			
Viral load (log₁₀ copies/mL)	2.356	0.125			

*Note:* Hosmer‐Lemeshow *p* = 0.106.

Abbreviations: BMI, body mass index; OR, odds ratio.

### 3.6 Correlation Between Metabolic and Inflammatory Parameters

**Figure 2:**
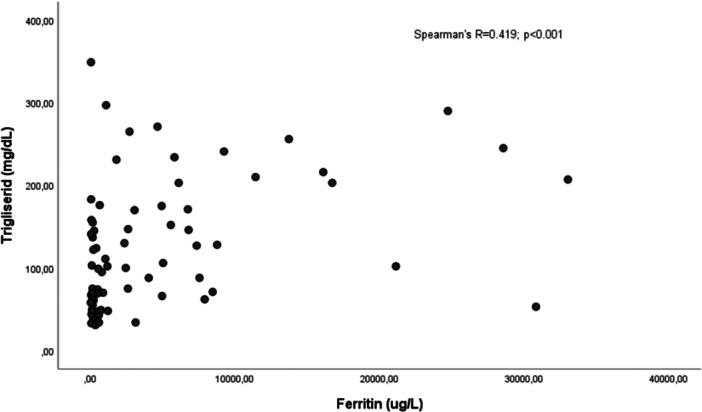
Spearman correlation analysis between serum triglyceride levels and ferritin.

**Figure 3:**
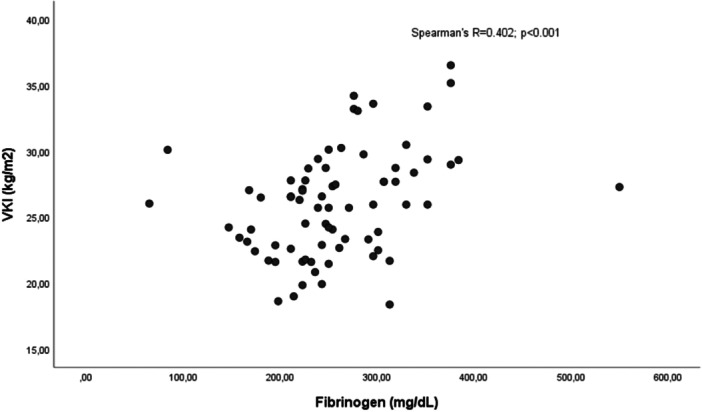
Spearman correlation analysis between Body Mass Index (BMI) and plasma fibrinogen levels.

**Figure 4:**
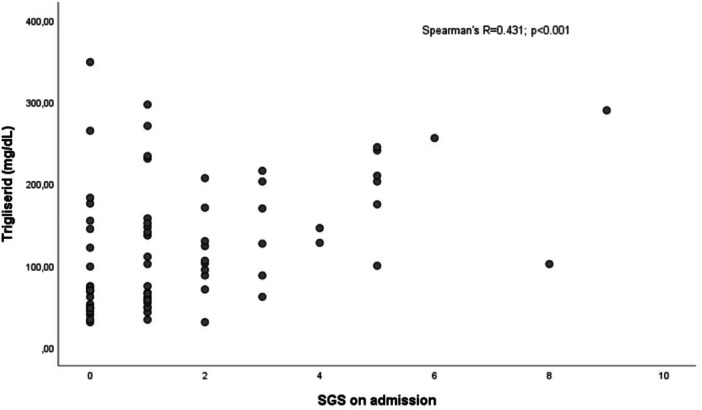
Spearman correlation analysis between serum triglyceride levels and the Severity Grading Score (SGS) at admission.

Correlation analysis revealed significant relationships between metabolic and inflammatory markers. Triglyceride levels showed moderate positive correlations with both ferritin levels and the SGS score, which reflects disease severity at admission (*p* < 0.001 for both). Additionally, a moderate positive correlation was observed between BMI and fibrinogen levels (*p* < 0.001; Figures 2, 3, 4).

## 4 Discussion

This study is one of the limited number of studies evaluating the impact of metabolic parameters and lipid profile on disease severity in patients with CCHF. Our findings demonstrate that BMI, FBG, and triglyceride levels, in particular, are significantly associated with disease severity in CCHF. These results suggest that not only viral factors but also the host's metabolic and vascular background play a crucial role in determining the clinical course of CCHF pathogenesis.

A critical consideration in interpreting our findings is whether the observed hyperglycemia reflects a pre‐existing metabolic condition or an acute stress‐induced response. In the absence of HbA1c data, a definitive distinction between undiagnosed chronic metabolic disorders and transient stress hyperglycemia cannot be made. Consequently, rather than establishing a definitive diagnosis of metabolic syndrome, the observed alterations were characterized as an ‘acute metabolic profile’ associated with CCHF. The elevated fasting blood glucose (FBG) levels, particularly in the moderate‐to‐severe disease group, likely represent a robust stress response mediated by the release of cortisol and catecholamines during viral sepsis.

However, several nuances in our data suggest a more complex pathophysiological role for glucose. Notably, the fact that FBG levels were significantly higher in the moderate‐to‐severe group compared to the mild group—despite both groups being evaluated during the same acute phase of infection—indicates that this hyperglycemia is not merely a universal, non‐specific stress response to the virus. Instead, it likely serves as a marker of the individual's underlying metabolic vulnerability or the specific intensity of the systemic insult. Most importantly, FBG remained an independent risk factor for disease severity in our multivariate model even after adjusting for viral load. This demonstrates that the metabolic derangement observed at admission is not merely a secondary reflection of high viremia, but rather a distinct pathophysiological process that independently contributes to or predicts the clinical outcome. These findings underscore the importance of early metabolic monitoring as a prognostic tool in CCHF management, regardless of the patient's baseline glycemic status. In this regard, unlike specialized immunological or molecular assays that may require sophisticated laboratory infrastructure and significant time, admission glucose and BMI are routine, cost‐effective, and provide immediate data during the initial triage of CCHF patients. While these parameters do not replace established biomarkers like viral load, they may serve as valuable complementary tools for early risk stratification, particularly in primary or secondary care centers where advanced diagnostic resources are limited.

Although the patient and control groups were comparable in terms of BMI and FBG, LDL‐C levels were found to be significantly suppressed in the patient group. This decline in serum lipids observed during acute infection is termed hypolipidemia of infection in the literature and is consistent with findings reported in general viral infections [10], as well as specifically in viral hemorrhagic fevers such as CCHF [11] and Dengue fever [12]. The underlying mechanism of this phenomenon involves the suppression of hepatic lipid synthesis and the increased consumption of lipoproteins during inflammatory processes, both of which are driven by the infection‐triggered pro‐inflammatory cytokine storm [10]. Therefore, this finding suggests that lipid profile alterations in CCHF patients serve as a dynamic indicator reflecting not only the patient's basal metabolic status but also the severity of the acute inflammatory response.

The role of metabolic parameters became more pronounced in analyses based on disease severity. The significantly higher levels of BMI, fasting blood glucose, and triglycerides in the moderate‐severe CCHF group indicate that the metabolic burden may negatively affect the clinical course of the disease. It is well established that metabolic disturbances, including obesity and impaired glycemic control, are characterized by a state of meta‐inflammation involving chronic low‐grade inflammation and impaired endothelial function [13].

In CCHF pathogenesis, where acute endothelial damage driven by the direct effect of the virus is the primary fatal mechanism [14], our results suggest that this chronic vascular fragility associated with pre‐existing metabolic irregularities combines with the acute endothelial destruction caused by the virus to create a two‐hit effect. This synergistic effect may explain why the clinical picture progresses more severely in patients with metabolic disorders.

In this context, the finding that fasting blood glucose levels had the highest discriminatory power in ROC analysis and served as an independent predictor in regression analysis is of particular importance. This condition, defined as stress hyperglycemia in the literature, is well known to worsen prognosis in acute infections [15]. Mechanistically, a hyperglycemic environment weakens the innate immune response by impairing neutrophil functions, while simultaneously triggering the production of pro‐inflammatory cytokines such as IL‐6 and TNF‐α [16]. More critically, high glucose concentrations lead to the degradation of the endothelial glycocalyx layer and increased vascular permeability by enhancing the production of Reactive Oxygen Species (ROS) [17]. Given that the vascular leakage process is central to CCHF pathophysiology, when combined with hyperglycemia‐induced endothelial toxicity, the hemorrhagic diathesis is exacerbated; thus, this mechanism emerges as one of the most critical factors determining disease severity.

In parallel, the association of triglyceride levels with severe disease highlights the strong interaction between inflammation and lipid metabolism. It is known in the literature that pro‐inflammatory cytokines inhibit lipoprotein lipase (LPL) activity, leading to hypertriglyceridemia [10]. The positive correlation detected between triglyceride and ferritin levels (r = 0.419) in our study validates this mechanism with clinical data. Ferritin is one of the most important indicators of macrophage activation and cytokine storm. Furthermore, the correlation of triglyceride levels with the SGS, which reflects disease severity at admission, suggests that this parameter can be considered not only a metabolic variable but also a surrogate marker of the acute inflammatory load and clinical severity in the patient.

Although BMI was not identified as an independent risk factor in regression analysis alone, its moderate discriminatory power in ROC analysis and significantly higher levels in the severe disease group are clinically noteworthy. The loss of statistical significance of BMI in multivariate analysis suggests that the impact of obesity on prognosis is exerted not directly, but indirectly through the metabolic derangements it induces (e.g., hyperglycemia, dyslipidemia). As described in the literature, visceral adipose tissue is an active endocrine organ secreting mediator such as IL‐6 and TNF‐α [18]. This chronic inflammatory background caused by obesity impairs the immune response to viral infections [19]. The positive correlation found between BMI and fibrinogen levels in our findings corroborates this pro‐thrombotic and pro‐inflammatory milieu. Since fibrinogen is both an acute‐phase reactant and a coagulation factor, this relationship provides strong evidence that increased adipose tissue may aggravate the coagulopathy and endothelial damage processes observed in CCHF. Therefore, BMI influences prognosis through these induced metabolic and hemostatic irregularities, even if not directly.

Our study has several limitations that should be acknowledged. First, the single‐center design and relatively modest sample size may restrict the generalizability of our findings to larger, more diverse populations. Second, the study population was unbalanced, with a smaller number of subjects in the healthy control group compared to the patient group. Although this imbalance may potentially limit the statistical power of certain comparisons, we employed robust non‐parametric and variance‐adjusted statistical tests to mitigate this effect and ensure the validity of our results.

Third, and perhaps most importantly, the lack of data regarding patients' pre‐infection metabolic status and HbA1c levels represents a significant limitation. Without longitudinal data or markers of long‐term glycemic control, it is challenging to definitively distinguish whether the observed metabolic disturbances particularly hyperglycemia reflect pre‐existing undiagnosed chronic conditions or acute stress‐induced responses triggered by the viral insult and subsequent inflammatory storm. Consequently, the metabolic alterations identified in this study are characterized as an ‘acute metabolic profile’ at the time of admission.

Despite these constraints, the systematic and statistically significant association between metabolic parameters and disease severity, which remained consistent even after adjusting for viral load, stands out as a major strength. These findings provide a solid basis for the prognostic utility of admission metabolic markers in the clinical management of CCHF.

## 5 Conclusion

This study demonstrates that key metabolic parameters, particularly fasting blood glucose and triglyceride levels, are not merely passive bystanders in the course of CCHF but are dynamic markers with a critical role in predicting disease severity. Our findings suggest that the acute endothelial damage caused by the virus synergizes with the host's metabolic background, creating a detrimental effect that worsens the prognosis. Therefore, the admission metabolic profile should be integrated into the initial assessment of CCHF patients. This approach, utilizing routine biochemical parameters, offers clinicians a cost‐effective and easily accessible perspective for early risk stratification and the identification of patients requiring more aggressive supportive care.

## Author Contributions

**Yasemin Çakır Kıymaz:** conceptualization, resources, writing – original draft, project administration. **Cihad Baysal:** investigation, data curation, methodology. **Caner Öksüz:** investigation, data curation. **Seyit Ali Büyüktuna:** formal analysis, visualization, software. **Nazif Elaldı:** formal analysis, supervision, writing – review and editing.

## Ethics Statement

This study was approved by the Sivas Cumhuriyet University Health Sciences Ethics Committee (Decision no: 2025‐06/45, Date: 12.06.2025).

## Consent

Written informed consent was obtained from the patients.

## Conflicts of Interest

The authors declare no conflicts of interest.

## Data Availability

The data that support the findings of this study are available from the corresponding author upon reasonable request. The data are not publicly available due to ethical restrictions and patient privacy concerns, as they contain sensitive clinical information.
